# Comparative Effects of Intermittent vs. Constant Ceftiofur Hydrochloride Exposure on *Staphylococcus aureus* In Vitro

**DOI:** 10.3390/antibiotics14070686

**Published:** 2025-07-06

**Authors:** Junli Wang, Chongyang Li, Fanxi Guo, Zugong Yu

**Affiliations:** Laboratory of Veterinary Pharmacology and Toxicology, College of Veterinary Medicine, Nanjing Agricultural University, Nanjing 210095, China; wjl7757@163.com (J.W.); 2020207011@stu.njau.edu.cn (C.L.); sensen12205@163.com (F.G.)

**Keywords:** Ceftiofur hydrochloride, adaptive evolution experiments, persistence, tolerance

## Abstract

Background/Objectives: Ceftiofur hydrochloride (CEF) is a third-generation cephalosporin widely used in cattle to treat various disease. The recommended dosage was 1.1 to 2.2 mg/kg BW for 3 to 5 consecutive days by intramuscular or subcutaneous injection. Incomplete treatment, overuse, or misuse, often observed in clinical practice, are major contributors to resistance development. This study aims to explore how different concentrations, durations, and dosing frequencies affect susceptibility and bactericidal efficacy of *Staphylococcus aureus* to optimize CEF dosage regimens. Methods: First, CEF was intermittently administered at 1/2 × minimum inhibitory concentration (MIC), 2 × MIC, 6 × MIC, and 100 × MIC for 30 cycles. Second, CEF was continuously administered for 48, 72, 96, 120, 144, and 168 h. Bacterial susceptibility, regrowth, survival rate, and the emergence of persisters or tolerant phenotypes were assessed. Genetic mutations were identified by whole-genome resequencing. Membrane permeability, integrity, and efflux pump activity were analyzed to elucidate the mechanism of CEF. Results: After 30 cycles, the MIC increased eight-fold in the 2 × MIC group. No significant MIC increase was found in other groups, but a progression from susceptibility to persistence and then to tolerance was observed in the 100 × MIC intermittent group. The survival rate increased both in the 2 × MIC and 100 × MIC groups. With continuous exposure to ≥6 × MIC over 120 h, strains were completely eradicated without MIC increase. Resistance-associated single-nucleotide polymorphism (SNP) mutations were detected only in strains of the 2 × MIC and 100 × MIC intermittent groups. CEF altered the membrane hydrophobicity, damaging membrane integrity after 30 cycles. Conclusions: These findings suggest that high-dose, prolonged exposure is more effective for eliminating *Staphylococcus aureus* and avoiding resistance, whereas intermittent dosing may promote persistence, tolerance, and resistance evolution.

## 1. Introduction

Ceftiofur hydrochloride (CEF) is a third-generation broad-spectrum cephalosporin widely used in cattle for treating respiratory infections, mastitis, acute metritis, and acute bovine interdigital necrobacillosis [1]. In 2018 alone, CEF consumption in China reached 260.42 tons [2]. Recommended treatment regimens require repeated administrations over 3 to 5 days, making therapeutic protocols relatively complex [3]. Consequently, despite its clinical importance, inappropriate dosing practices—such as incomplete courses, overuse, or misuse—are common [4]. These practices result in variable drug concentrations and exposure durations at the target set, which can alter bacteria susceptibility and promote resistance development [5]. Furthermore, suboptimal dosing may induce the transition of susceptible bacteria cells into persister cells and facilitate cross-resistance to human-use cephalosporins like ceftriaxone, with resistance rates approaching 99% among ceftiofur resistant isolates [6,7]. These challenges highlight the urgent need to optimize CEF dosing regimens to improve therapeutic efficacy while minimize the emergence of resistance.

Previous studies have suggested that continuous infusion of time-dependent antibiotics may be more cost effective and potentially reduce resistance development compared to intermittent administration [8,9]. However, other studies indicate that constant and prolonged drug exposure can promote susceptible bacteria to resistance through the evolution of persistence or tolerance [10]. Persistence refers to the ability of a subpopulation to survive lethal concentrations of antibiotics without changes in the minimum inhibitory concentration (MIC), typically exhibiting a biphasic killing curve [11]. Tolerance is described as a population-level phenomenon that enables bacteria to survive antibiotic concentrations exceeding the MIC without acquiring genetic resistance or altering the MIC. Persistence cells can also be regarded as a subset of tolerance cells, occurring in a small fraction of the population. When 100% of the population exhibits persistent behavior, the phenotype is essentially indistinguishable from tolerance [12].

*Staphylococcus aureus (S. aureus)* is a major pathogen responsible for both mastitis and endometritis in dairy cows, leading to reduced fertility in cows and significant economic losses in the dairy industry [13,14]. Notably, *S. aureus* strains isolated from dairy cows have exhibited high levels of resistance to ceftiofur in certain regions [15], raising concerns about the long-term efficacy of cephalosporin antibiotics. To address this challenge, optimizing the dosing regimens of CEF is considered a promising strategy.

Like other β-lactam antibiotics, CEF exerts its bactericidal effect by binding to penicillin-binding proteins (PBPs), leading to bacterial lysis and death. However, bacteria resistance to β-lactams arise through the production of β-lactamase, modifications of target site, altered membrane permeability, or active efflux mechanisms [16]. The influence of CEF exposure on resistance development and related phenotypes in *S. aureus* remains poorly understood.

Therefore, this study aimed to investigate how variations in CEF concentration, exposure duration, and dosing frequency affect the susceptibility and bactericidal activity against *S. aureus*, as well as the emergence of persister cells, tolerance, and resistance. Additionally, we investigated whether CEF exposure influences membrane permeability, membrane integrity, or efflux pump activity, with the goal of identifying novel targets for antimicrobial intervention and informing the rational use of CEF in veterinary practice to enhance therapeutic efficacy and delay resistance development.

## 2. Results

### 2.1. Results of Intermittent Exposure to CEF at 1/2 × MIC, 2 × MIC, 6× MIC, and 100 × MIC

The minimum inhibitory concentration (MIC), minimum bactericidal concentration (MBC), and mutant prevention concentration (MPC) of the parental strain RY-Z5 (0 cycle) were 0.5 µg/mL, 1 µg/mL, and 4.8 µg/mL, respectively.

**Figure 1:**
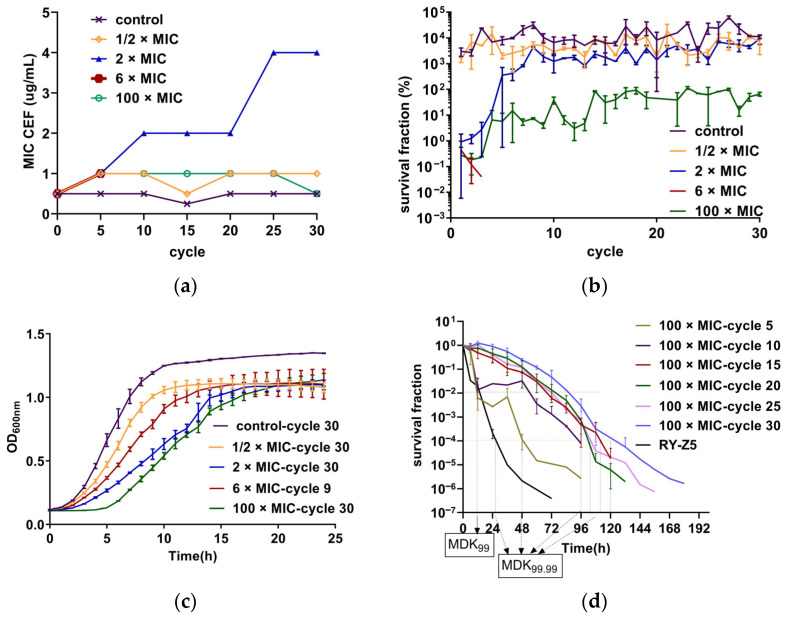
(**a**) The MICs of CEF were determined every five cycles using the micro-broth dilution method according to CLSI guidelines. MIC values represent the highest concentration reproducibly measured from three independent replicates (n = 3). (**b**) Survival fractions of *S. aureus* RY-Z5 strains exposed to intermittent CEF treatment across 30 cycles. Five parallel evolutionary lineages were maintained at each CEF concentration. Each data point represents the mean ± SD of three independent experiments (n = 3); the untreated group served as the control. (**c**) Growth curves of endpoint strains after 30 exposure cycles. Each curve represents the mean ± SD from three independent experiments with six technical replicates per group (n = 3). (**d**) Time–killing curves of evolved strains exposed to 50 ug/mL CEF (100 × MIC). RY-Z5 represents the unexposed parental strain. Bacteria survival was quantified by CFU counting at designated timepoints and expressed as the ratio CFU (n h)/CFU (0 h). Values represented correspond to the mean ± SD of three measurements (n = 3). MDK_99_ and MDK_99.99_ were estimated based on survival fraction thresholds (1% and 0.01%, respectively).

An in vitro adaptive evolution experiment was performed to investigate the bactericidal efficacy and impact on resistance evolution of CEF under intermittent administration at different concentrations. After 30 cycles, the MIC increased eight-fold in the 2 × MIC group (1 µg/mL) compared to the parental strain, shifting the strain from susceptible to non-susceptible (Figure 1a). And the initial survival rate was low but gradually increased, nearly equivalent to the control group by the fifth cycle, suggesting a reduced bactericidal effect (Figure 1b). No significant increase in the MIC was found at other concentrations (no more than 2 times). The bacterial strains in the 6 × MIC group were completely eradicated after 7–9 cycles, which is quite different from others even under a high concentration of antibiotic. “Completely eradicated” herein refers to the absence of bacterial regrowth 48 h after drug removal. The survival rate of strains in the 1/2 × MIC group was similar to the untreated group (control group). In the 100 × MIC group (50 µg/mL), the survival rate was low initially too but increased gradually, stabilizing around 10% after the fifth cycle, indicating that a small subset of bacteria could still escape from drug killing (Figure 1b). Furthermore, the analysis of the growth curve showed that after 30 cycles, strains exposed to 50 µg/mL CEF displayed a notably prolonged lag phase compared to those treated under other conditions (Figure 1c). This extended lag phase may represent a persistence-like adaptation that enables survival under sustained antibiotic pressure [17].

We performed detailed time–killing experiments using a CEF concentration of 50 µg/mL (100 × MIC) to assess the presence of persistence and tolerance [18]. The results are presented in Figure 1d. The minimum duration required to kill 99% of bacterial cells in the population (MDK_99_) and the MIC values of strains in the 5th and 10th treatment cycles remained the same as those of the parental strain. However, the minimum duration to kill 99.99% of bacterial cells in the population (MDK_99.99_) is significantly prolonged, and biphasic killing curves were observed, indicating the presence of persisters in these cycles. In contrast, strains from the 15th, 20th, 25th, and 30th cycle exhibited increased MDK_99_ values compared to the parental strain, with unchanged MIC values but without biphasic killing kinetics. These findings suggest a shift from persistence to tolerance during repeated intermittent high-dose exposure.

### 2.2. Results of Single Prolonged Exposure to CEF for 48, 72, 96, 120, 144, and 168 h

**Figure 2:**
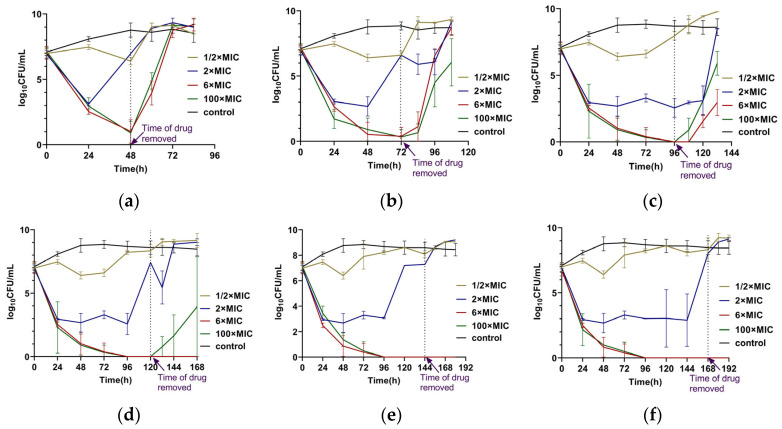
Single-dose administration of CEF with different concentrations and different durations: (**a**) For 48 h. (**b**) For 72 h. (**c**) For 96 h. (**d**) For 120 h. (**e**) For 144 h. (**f**) For 168 h. The dashed line represents the timepoint at which the drug was removed. Each experiment was performed in triplicate and in parallel. Dots on the lines represented the mean ± SD values of all the experiments on a log scale (n = 3).

Single-dose administrations of CEF with different durations and concentrations were conducted. The results showed that the administration of CEF at 6 × MIC and 100 × MIC resulted in similar bactericidal kinetics (Figure 2). Treatment with 2 × MIC exhibited a bactericidal effect initially but failed to achieve complete bacterial eradication, which is similar to those of intermittent administration. No bacterial regrowth was observed after drug removal when bacteria were exposed to 6 × MIC or 100 × MIC for ≥120 h. In contrast, at other concentrations or exposure durations, the strains regrew to the stationary phase within 24 h after drug withdrawal, as determined by CFU counts. Notably, the MIC values of the surviving bacteria at the endpoint remained at 0.5 µg/mL, identical to those of the parental strain, indicating that no resistance had developed.

### 2.3. Results of Genome Resequencing Analysis

To investigate genetic changes during adaptive evolution, four endpoint strains from the evolution experiments were subjected to whole-genome resequencing and compared to their parental strains. These included strains from the 30th cycle at 1/2 × MIC, 2 × MIC, and 100 × MIC of CEF exposure, as well as the 9th cycle at 6 × MIC. The results are summarized in Table 1. Compared with parental strains, all samples exhibit a deletion mutation in the intergenic region (IGR). The strain from 6 × MIC, 9th cycle, has another deletion mutation in the coding sequence (CDS) region, and annotation analysis revealed that this region is related with coding Methionyl-tRNA synthetase. The single-nucleotide polymorphism (SNP) mutations of the genome observed were classified as nonsynonymous, synonymous, and stoploss, all located within CDS regions. The strain 1/2 × MIC, 30th cycle, has one stoploss SNP mutation. The strain 2 × MIC, 30th cycle, has five SNP mutations: four are nonsynonymous, and one is synonymous. The strain 6 × MIC, 9th cycle, has two nonsynonymous SNP mutations. The strain 100 × MIC, 30th cycle, exhibited six SNP mutations: two stoploss, two synonymous, and two nonsynonymous. Details of all SNP mutations are provided in Table 2. In addition, structural variations (SV)s in the four strains were detected, and the results are summarized in Table 3.

### 2.4. Results of Membrane Permeability, Membrane Integrity, and Efflux Pump Assays

**Figure 3:**
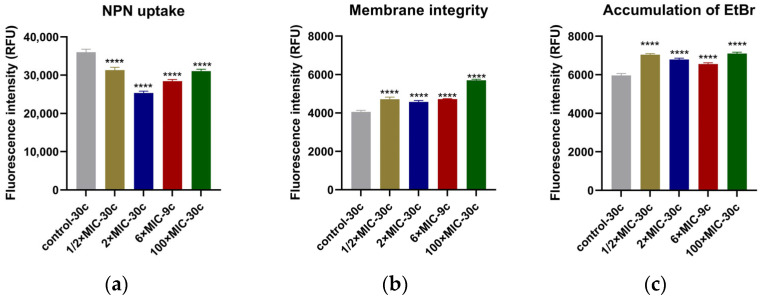
Results of five endpoint strains from the evolution experiments analyzed for the membrane permeability, membrane integrity, and accumulation of EtBr. (**a**) Strains treated with CEF showed lower NPN fluorescence values compared to untreated group (control-30 c); (**b**) CEF binds with PBPs on the cell wall and damages the membrane integrity, causing increased fluorescence intensity of PI uptake; (**c**) CEF directly or indirectly inhibits the efflux pump activity with no dose-dependent manner, causing EtBr accumulation within the cell and a corresponding increase in fluorescence intensity. All the tests were performed in triplicate, and all the data are presented as the mean ± SD; the significances were determined by one-way analysis of variance (ANOVA). **** *p* < 0.001.

The hydrophobic probe of 1-N-phenyl-naphthylamine (NPN) fluoresces weakly in aqueous environments but emits strong fluorescence when it binds to the hydrophobic region of the cell membrane, reflecting changes in membrane permeability [19]. Compared to the untreated strains at the 30th cycle (control-30 c), the NPN fluorescence intensity of all CEF-treated groups was significantly lower (*p* < 0.001) (Figure 3a), traditionally indicating a reduction in membrane permeability. The fluorescence intensity of propidium iodide (PI) of all the treated groups was significantly higher than the untreated group (*p* < 0.001) (Figure 3b), suggesting that after 30 cycles of drug exposure, the membrane integrity of strains was damaged.

Under normal conditions, the fluorescent probe of ethidium bromide (EtBr) emits only weak fluorescence. However, it can enter cells through passive diffusion and bind to intracellular DNA, producing strong fluorescence. The efflux pump actively transports EtBr out of the cell. When the activity of the efflux pump is inhibited, EtBr begins to accumulate inside the cell, causing the fluorescence intensity to gradually increase. The results showed that at the end of the cycles, strains of all the treated groups exhibited significantly higher EtBr fluorescence intensity compared to the untreated control (*p* < 0.001) (Figure 3c), indicating intracellular accumulation of EtBr.

## 3. Discussion

This study investigated the effects of intermittent and constant administration of CEF at different concentrations on the bacterial sensitivity, survival rate, regrowth rate, and evolution of drug resistance in *S. aureus*. Most previous studies have selected a constant sub-MIC [20,21,22], stepwise increasing concentrations starting from the sub-MIC [23,24], a constant lethal concentration [25], or a periodic constant lethal concentration to study the evolution of resistance [26]. Some studies have also investigated intermittent exposure to varying drug concentrations over different durations. In our study, we choose four different concentrations: sub-MIC (1/2 × MIC), 2 × MIC, 6 × MIC, and 100 × MIC in both constant and intermittent administrations. First, maintaining plasma concentrations above 0.2 µg/mL (t > 0.2) is critical for CEF in clinical [27]. Second, MBC and MPC are both crucial reference points in resistance development. Therefore, the selected concentrations for this study were 0.25 µg/mL (sub-MIC, 1/2 × MIC), 1 µg/mL (>MBC, 2 × MIC), 3 µg/mL (6 × MIC, between MBC and MPC), and 50 µg/mL (MPC, 100 × MIC).

In our study, the MICs of strains treated with the 1/2 × MIC concentration of CEF remained the same as their parental strains, regardless of whether the exposure was via periodic or continuous administration. This finding contrasts with the findings of Santo’s study [21], where the *Acinetobacter baumannii* populations developed significantly increased MICs after 7 days of continuous exposure to a sub-MIC concentration of ciprofloxacin, eventually evolving resistance after 12 days. However, our results are consistent with Ahmed’s study [20], who reported no significant MIC changes following 6 cycles of either periodic or continuous sub-MIC exposure. This discrepancy may be due to insufficient selective pressure exerted by sub-MIC concentrations, as well as species-specific differences in mutation mechanisms. A previous study has suggested that antimicrobial resistance mutations are more likely to emerge when drug concentrations fall within the MSW, with concentrations between the MIC and MPC [28]. In our study, both 1 µg/mL (2 × MIC) and 3 µg/mL (6 × MIC) fall within the MSW of CEF. However, after 30 cycles of intermittent exposure to 1 µg/mL of CEF, the MIC of the evolved strain increased 8-fold. In contrast, strains exposed to 3 µg/mL for 7 to 9 cycles were completely eradicated, with no regrowth observed after drug removal. These results differ from previous reports. For example, Usui’s study reported that 0.1% of planktonic *Escherichia coli* survived after 7–10 cycles of periodic exposure to 5 × MIC of amikacin [26]. Similarly, Michiels’ study found that persistent *S. aureus* emerged following repeated exposure to aminoglycosides at concentrations exceeding 6 × MIC [29]. One explanation for these differences may lie in the pharmacodynamic properties of the antibiotics used. The model drugs in these studies—ciprofloxacin, amikacin, and other aminoglycosides—are concentration-dependent antibiotics, whereas CEF is a time-dependent β-lactam. To date, limited data are available on resistance evolution under time-dependent antibiotic intermittent exposure. Our findings may provide new insights that fill this gap in understanding. Furthermore, the MSW concept applies to drugs that are not lethal and should be modified when investigating resistance caused by efflux, target alteration, or the suppression of inactivating enzyme production [30].

Research has shown that persistent bacteria can escape from high lethal concentrations of antibiotics, and tolerance enables bacterial cells to survive exposure to concentrations that would otherwise be lethal [17]. In this study, both persistence and tolerance were observed when bacteria were subjected to periodic lethal antibiotic exposure. Our results suggest that tolerance may have emerged following the development of persistence, a pattern that aligns with the sequential transition described by Hu et al., where bacteria sequentially exhibited persistence, then tolerance, and eventually resistance [31]. As shown in Figure 1a and Figure 1d, the MIC and MDK_99_ values of strains in the 5th and 10th cycles were identical to those of the parental strain RY-Z5, but the MDK_99.99_ values increased. Also, the significantly biphasic killing curves suggested the development of a persistent subpopulation. After the 10th cycle, an increased MDK_99_ was observed, which may indicate the development of tolerance. Furthermore, we found that the lag times of bacteria under 50 µg/mL CEF exposure were longer than those under other conditions (Figure 1c), potentially reflecting an adaptive adjustment of single-cell lag-time distributions to better match the antibiotic exposure intervals, as proposed by Fridman et al. [17]. While these findings are consistent with a possible sensitivity–persistence–tolerance progression, we acknowledge that direct mechanistic confirmation is lacking, and alternative explanations cannot be excluded. Further studies integrating single-cell tracking, transcriptomics, and genetic manipulation will be required to delineate the precise evolutionary trajectory and underlying molecular mechanisms.

Persisters play a critical role in the recalcitrance and relapse of bacterial infections, and they can facilitate the emergence of antibiotic resistance during treatment [11]. In our study, constant antibiotic administration was more effective than intermittent administration in reducing or inhibiting the formation of persisters. Specifically, when *S. aureus* was continuously exposed to CEF at concentrations ≥ 6 × MIC for more than 120 h, no bacteria survival or regrowth was observed after drug removal. These results are consistent with previous clinical findings that continuous infusion at concentrations exceeding 4 × MIC is more effective than intermittent administration [32]. As a time-dependent antibiotic, CEF requires a higher concentration and prolonged exposure to maintain efficacy and achieve complete bacterial eradication without MIC increase or tolerance development. And constant administration of CEF at concentrations above 6 × MIC for more than 120 h is more effective than intermittent dosing. However, some reports suggest that persistent intramammary administration of CEF can lead to an increased prevalence of resistant bacteria within the gut microbiota [33]. Moreover, although direct evidence of ceftiofur-induced nephrotoxicity is limited, high doses of related β-lactam antibiotics have been associated with renal injury in rats [34]. Therefore, optimizing dosage regimen should balance antimicrobial efficacy with the potential risk of toxicity.

In this study, the analysis of membrane permeability and integrity revealed notable changes after 30 treatment cycles. Specifically, NPN fluorescence values were lower in the CEF-treated group compared to untreated group. This phenomenon may be attributed to three possible mechanisms: first, CEF binding to PBPs on the *S. aureus* cell wall, leading to cell lysis and death [35]; second, adaptive bacterial responses that alter membrane permeability, thereby reducing NPN probe uptake [16]; and last, when the bacterial cell membrane undergoes remodeling or sustains severe damage, NPN may escape from the hydrophobic regions of the membrane, resulting in decreased fluorescence intensity [36]. Further analysis of membrane integrity showed increased PI fluorescence, indicating damage to the cellular structure. Additionally, efflux pump analysis showed increased EtBr accumulation. This may result from three factors: first, CEF-induced membrane damage, allowing more EtBr entry and binding to intracellular DNA; second, the inhibition of efflux pump activity after repeated CEF exposure, reducing active export of EtBr; and third, differential physicochemical properties of the probes—EtBr is hydrophilic, while NPN is hydrophobic—suggesting that altered membrane hydrophobicity may decrease NPN uptake while simultaneously enhancing EtBr retention. Together, these findings suggest that CEF may alter membrane surface hydrophobicity, compromise membrane integrity, and impair efflux pump function, thereby contributing synergistically to its bactericidal activity against *S. aureus*.

Whole-genome resequencing of strains subjected to intermittent exposure to CEF revealed adaptive mutations. All treated strains exhibit a deletion mutation in the intergenic region located between the genes encoding the 50S ribosomal protein L19 and a hypothetical protein, which may affect the expression of adjacent genes and potentially influence antibiotic resistance [37]. In the strain treated with 2 × MIC CEF, four nonsynonymous mutations were identified. Two were in the genes encoding PBP1, a protein associated with resistance to cephalosporin and amoxicillin of *S. aureus* [38,39]. Another occurred in a gene encoding a signal transduction histidine kinase, which is related to resistance to vancomycin and daptomycin [40]. Additionally, a mutation was found in the QacA gene, which encodes proton-coupled efflux of mono- and divalent cationic antibacterial compounds, and mutations in QacA gene are associated with resistance and tolerance to cationic antibacterial compounds like chlorhexidine in *S. aureus* [41,42]. These mutations may also contribute to reduced susceptibility to CEF. In the strain exposed to 6 × MIC µg/mL CEF, two nonsynonymous mutations were identified, neither of which is directly associated with resistance, unlike the mutations observed in the 2 × MIC or 100 × MIC groups. One mutation was located in a gene encoding a hypothetical protein, and the other in the mannose-6-phosphate isomerase gene. The latter is a key enzyme in the D-mannose pathway, linking mannose metabolism and cell wall biosynthesis. Disruption of this pathway has been associated with cell wall defects and increased susceptibility to β-lactam antibiotics [43]. Therefore, the mutation in the mannose-6-phosphate isomerase gene may partially explain the bacterial eradication observed after 7–9 cycles of 6 × MIC CEF exposure. The mutation in the hypothetical protein gene may further exacerbate this effect, although its exact role remains unclear. In strains exposed to 100 × MIC CEF for 30 cycles, two nonsynonymous SNP mutations were identified. One was located in the prolyl-tRNA synthetase gene, associated with halofuginone resistance both in vitro and in vivo [44], and the other one was also located in QacA gene. Our findings suggest that mutations in these two genes may also contribute to the tolerance of *S. aureus* to CEF.

However, all experiments in this study were conducted under constant concentrations in vitro, which differ significantly from the dynamic drug concentration fluctuations that occur in vivo. Therefore, further studies using pharmacokinetic–pharmacodynamic (PK/PD) models or animal models are necessary to validate the efficacy and safety of different dosage regimens.

## 4. Materials and Methods

### 4.1. Bacterial Strains, Culture Conditions, and Reagents

The *S. aureus* strain RY-Z5 used in this study was isolated from an endometrial swab of a cow diagnosed with metritis in Zhengzhou, China. Initial identification was based on colony morphology, and the strain was further confirmed by 16S rDNA gene sequencing using the primers forward (F) 5′-GGACGACATTAGACGAATCA-3′ and reverse (R) 5′-CGGGCACCTATTTTCTATCT-3′. *S. aureus* ATCC 29213 was purchased from Hope Bio-Technology Co., Ltd. All bacterial strains were cultured at 37 °C in Tryptic Soy Broth (TSB) or on Tryptic Soy Agar (TSA) plates.

CEF was purchased from Amicogen (Jining, China), Lot No. PS 077-2304071. A 1280 µg/mL CEF stock solution was prepared by accurately weighing 0.0348 g of CEF powder, dissolving it in methanol, and adjusting the final volume to 25 mL. The stock solution was aliquoted into 2 mL EP tubes and stored at −20 °C for subsequent experiments.

### 4.2. MIC, MBC, and MPC Determination

The MIC of CEF for each sample needed were determined using the micro-broth dilution method in accordance with Clinical and Laboratory Standards Institute (CLSI) guidelines. *S. aureus* ATCC 29213 was used for quality control. Briefly, *S. aureus* cultures were diluted to a final inoculum of approximately 5 × 10^5^ CFU/mL in Mueller–Hinton (MH) broth. Serial two-fold dilutions of CEF were prepared in 96-well microtiter plates to achieve final concentrations ranging from 0.065 µg/mL to 64 µg/mL. After 18–24 h of incubation at 37 °C, the MIC was defined as the lowest antibiotic concentration that completely inhibited visible bacterial growth.

To determine the minimum bactericidal concentration (MBC), 100 µL aliquots from wells showing no visible growth were plated on MH agar and incubated at 37 °C for 24 h. The MBC was defined as the lowest concentration of CEF that can kill 99.9% of the bacteria in the culture medium, corresponding to a ≥3 log_10_ reduction in CFU/mL compared to the initial inoculum [45].

To determine the mutant prevention concentration (MPC), 100 mL of *S. aureus* RY-Z5 was incubated overnight at 37 °C in TSB. The bacterial concentration was estimated to be ≥3 × 10^8^ CFU/mL based on the optical density at 600 nm. The cultures were then concentrated by centrifugation at 6000 × g for 10 min and resuspended in 3 mL of MH broth [46]. A 100 µL aliquot, containing ≥1 × 10^10^ CFU, was evenly spread onto MH agar plates containing various concentrations of CEF, including MIC, 2 × MIC, 4 × MIC, 8 × MIC, 16 × MIC, 32 × MIC, and 64 × MIC. The plates were incubated at 37 °C for 72 h, and colony formation was assessed every 24 h. The lowest drug concentration at which no colonies were observed after 72 h of incubation was designated as the provisional mutant prevention concentration (MPCpr). Based on the MPCpr, a new series of MH agar plates were prepared with CEF concentrations reduced linearly by 20%. The former procedure was repeated using the same high-density bacterial suspension. The lowest concentration at which no colony growth was observed after another 72 h of incubation was determined as the final mutant prevention concentration (MPC). All experiments were performed in triplicate, with four parallel samples per replicate.

### 4.3. Intermittent CEF Exposure with Different Concentrations by In Vitro Adaptive Evolution Experiments

The method was adapted from former studies, with modifications [17,26]. Briefly, 30 µL of overnight-cultured RY-Z5 was diluted in fresh TSB to an initial inoculum of 10^7^ CFU/mL (30 µL culture into 3 mL TSB). The cultures were supplemented with CEF at final concentrations of 0, 0.25, 1, 3, and 50 µg/mL and then incubated at 37 °C with shaking at 200 rpm for 12 h. The drug was then removed by washing twice with 0.3 mL of fresh TSB (centrifugation at 8000 rpm, 8 min, 25 °C). The bacterial pellet was resuspended in 3 mL of TSB and incubated overnight to complete one cycle. This process—overnight culture, dilution, CEF exposure, antibiotic washout, and regrowth—constituted one complete cycle and was repeated for 30 cycles. CFU counts were performed before and after antibiotic exposure to calculate the survival rate. Samples of every 5 cycles and endpoints were stored at −80 °C in 60% glycerol for further analysis. The experiments were repeated three times, with three parallel samples per repeat.

### 4.4. Single-Dose Administration of CEF with Different Durations and Concentrations

In total, 30 µL of overnight-cultured RY-Z5 was inoculated into 3 mL of TSB to an initial inoculum of 10^7^ CFU/mL. The cultures were added with different concentrations of CEF and incubated at 37 °C with shaking at 200 rpm for different durations (48 h, 72 h, 96 h, 120 h, 144 h, and 168 h). After removing the drug by washing twice in TSB (centrifugated at 8000 rpm, 8 min, 25 °C), the culture was resuspended in 3 mL of TSB and incubated till the bacterial regrowth reached the stationary stage or no growth was observed after 48 hours of incubation. Bacterial counts were performed every 12 h, and endpoint strains were stored at −80 °C in 60% glycerol for further analysis. The experiments were repeated three times, with three parallel samples per repeat.

### 4.5. CFU Count and Survival Rate

The sample was 10-fold serially diluted in TSB medium, and 10 µL of each dilution was plated on column of a TSA square plate. After incubated at 37 °C for 24 h, the visible colonies on the plate were counted. Only colonies between 30 and 300 were recorded as reliable results. The CFU count is calculated by multiplying the number of colonies by the dilution factor.

CFU count = Number of colonies × Dilution factor × (1000 µL/volume plated)

The survival rate of each cycle was calculated using the following equation:

survival% = CFU_aftertreatment_/CFU_beforetreatment_ × 100%

### 4.6. Growth Curve Determination

The overnight-cultured endpoint strains were diluted to 10^5^ ~ 10^6^ CFU/mL with fresh TSB, and 200 μL of the dilution was added to each well of 96-well plate [26]. The plate was incubated in a spectrophotometer at 37 °C for 24 h. OD_600 nm_ was measured hourly. Growth curves were plotted with time on the *x*-axis and OD_600 nm_ on the *y*-axis. Experiments were performed in triplicate, with six parallel wells per replicate.

### 4.7. Time–Kill Assay

To evaluate bacterial persistence and tolerance, time–kill assays were performed using a high concentration of CEF [18,31]. The overnight-cultured medium was diluted to a density of 10^6^–10^7^ CFU/mL and supplemented with CEF at a final concentration of 50 µg/mL. The bacterial suspensions were incubated at 37 °C with shaking at 200 rpm. Samples were collected every 12 h to perform the CFU count. Survival fractions were plotted over time to assess bactericidal activity. MDK_99_ refers to the minimum duration required to kill 99% of bacterial cells in the population, equal to 1% survival fractions, and MDK_99.99_ defined as the minimum duration to kill 99.99% of bacterial cells in the population, equal to 0.01% survival fractions. Each experiment was conducted in triplicate, with three parallel samples per replicate.

### 4.8. Whole-Genome Resequencing Analysis

Whole-genome resequencing was performed to identify gene mutations under different concentrations of periodic administration. The endpoint strains under intermittent exposure to CEF were revived and incubated at 37 °C till the bacteria reached the exponential phase. The medium was removed, and incubations were sent to Shanghai Biozeron Biothchnology Co., Ltd. (Shanghai, China) for whole-genome resequencing. DNA was extracted using a Bacterial DNA Kit (OMEGA, Guangzhou, China) according to the manufacturer’s instructions. Sequencing was performed on an Illumina NovaSeq 6000 platform with paired-end reads. The raw paired-end reads were trimmed and quality controlled by Trimmomatic (version 0.36). The clean sequencing reads were aligned to *S. aureus* RY-Z5 genome sequence using BWA software. Sequence Alignment Map (SAM) files were imported into SAMtools for sorting and merging, and Picard (version 1.92) was used to remove duplicated reads. The sequencing depth and coverage were calculated based on the alignments by custom perl scripts. SNPs were detected by the valid BAM file, and short InDel were identified by GATK (version 4.1.2.0) with the “HaplotypeCaller” function. Structure variations (SVs) were identified using BreakDancer. The identified variants were annotated to determine their potential functional consequences using ANNOVAR (http://www.openbioinformatics.org/annovar/, accessed on 17 July 2017).

### 4.9. Membrane Permeability, Membrane Integrity, and Efflux Pump Assay

The membrane permeability was analyzed with fluorescent probe 1-N-phenyl-naphthylamine (NPN, Macklin, Shanghai, China) [47,48]. The endpoint strains under intermittent administration of CEF were revived and incubated at 37 °C to the exponential phase. Strains were collected, washed, and resuspended in 5 mM HEPES buffer (pH 7.2) containing 5 mM glucose (HEPES-g) to an OD_600 nm_ of 0.5. Then, 1 mmol/L NPN solution was added to the bacterial suspension at a final concentration of 10 µmol/L. After incubating for 30 min in the dark at 37 °C, the fluorescence value was measured by a fluorescence spectrophotometer at excitation and emission wavelengths of 350 nm and 420 nm, respectively. The experiments were performed in triplicate, with four parallel samples per replicate.

A modification method was used to assay the membrane integrity of cycle strains [49,50]. Revived and incubated to the exponential phase, samples were washed and resuspended in 5 mM HEPES-g and adjusted to an OD_600 nm_ of 0.5. Then, 1 mmol/L propidium iodide (PI, Shanghai yuanye Bio-Technology Co., Ltd. Shanghai, China.) was added to a final concentration of 10 µmol/L. After incubating for 30 min in the dark at 37 °C, the fluorescence value was measured at excitation/emission wavelengths of 535 nm/615 nm. The experiments were performed in triplicate, with four parallel samples per replicate.

Efflux pump activity was assessed using a modified method [51]. Bacteria were incubated to the exponential phase and adjusted to an OD_600 nm_ of 0.5 in 5 mM HEPES-g. Ethidium bromide (EtBr, Macklin, Shanghai, China) was added to the bacterial suspension at a final concentration of 1 µmol/L, and the mixture was incubated in the dark at 37 °C for 30 min. The fluorescence value was then measured at an excitation wavelength of 525 nm and emission wavelength of 600 nm. The experiments were performed in triplicate, with four parallel samples per replicate.

### 4.10. Statistical Analysis

Statistical analyses were performed using GraphPad Prism version 9.0. One-way analysis of variance (ANOVA) was used to test for statistical significance (*p* < 0.001).

## 5. Conclusions

In conclusion, constant administration of CEF at concentrations above 6 × MIC for more than 120 h proved to be more effective than intermittent dosing, with no bacterial regrowth or resistance observed. In contrast, under intermittent administration at 2 × MIC, strains were more likely to develop resistance after 30 cycles, and antibiotic tolerance evolved following persistent exposure to high lethal concentrations. Moreover, after 30 cycles of intermittent treatment, CEF was found to alter membrane hydrophobicity and disrupt membrane integrity. A single prolonged administration of CEF may simplify clinical procedures, reduce the risk of underdosing or overdosing, improve therapeutic outcomes, and help minimize the emergence of antimicrobial resistance.

## Figures and Tables

**Table 1:** Statistics of sample sequencing data and comparison results with their parental strains.

Sample	Clean Data	Q20% ^1^	Q30% ^2^	Mapped Reads	Mapped Rate (%)	Coverage (%)	Aver-Dep ^3^
1/2 × MIC	7,590,116	98.56	96.6	7,570,381	99.74	99.97	408.0
2 × MIC	7,721,716	98.57	96.67	7,690,056	99.59	99.97	414.0
6 × MIC	8,540,206	98.52	96.45	8,516,293	99.72	99.97	458.9
100 × MIC	7,654,960	98.54	96.52	7,636,588	99.76	99.97	411.6

^1^ Q20% refers to the proportion of bases with a Phred quality score greater than 20, reflecting overall base-calling accuracy. ^2^ Q30% refers to the proportion of bases with a Phred quality score greater than 30, representing higher-confidence base calls. ^3^ Aver-dep (average depth) is calculated as the total number of bases from all reads divided by the genome size, representing the average coverage across the genome.

**Table 2:** SNP mutations of endpoint strains from the evolution experiments.

Sample	Type	Annotation of Coding Gene
1/2 × MIC	stoploss	
2 × MIC	nonsynonymous	Multimodular transpeptidase-transglycosylase/Penicillin-binding protein 1A/1B (PBP1)
	nonsynonymous	Multimodular transpeptidase-transglycosylase/Penicillin-binding protein 1A/1B (PBP1)
	nonsynonymous	Signal transduction histidine kinase
	synonymous	RGD-containing lipoprotein
	nonsynonymous	Drug resistance transporter, EmrB/QacA subfamily
6 × MIC	nonsynonymous	Hypothetical protein
	nonsynonymous	Mannose-6-phosphate isomerase
100 × MIC	nonsynonymous	Prolyl-tRNA synthetase, bacterial type
	stoploss	Phosphoesterase, DHH family protein
	stoploss	Phosphoesterase, DHH family protein
	synonymous	LSU m5C1962 methyltransferase RlmI
	synonymous	Membrane component of multidrug resistance system
	nonsynonymous	Drug resistance transporter, EmrB/QacA subfamily

**Table 3:** SVs of endpoint strains from the evolution experiments.

Sample	Inter-Chromosomal Translocation	Deletion	Insertion	Inversion	Intra-Chromosomal Translocation	Total
1/2 × MIC	42	0	6	0	2	50
2 × MIC	43	1	1	0	4	49
6 × MIC	44	1	3	0	5	53
100 × MIC	43	1	1	0	3	48

## Data Availability

Data are contained within the article.

## Abbreviations

The following abbreviations are used in this manuscript:

CEF	Ceftiofur hydrochloride
BW	Body weight
*S. aureus*	*Staphylococcus aureus*
PBPs	Penicillin-binding proteins
MIC	Minimum inhibitory concentration
MBC	Minimum bactericidal concentration
MPC	Mutant prevention concentration
MDK_99_	Minimum duration required to kill 99% of bacterial cells in the population
MDK_99.99_	Minimum duration required to kill 99.99% of bacterial cells in the population
CDS	Coding sequence
SNP	Single-nucleotide polymorphism
SV	Structural variation
NPN	1-N-phenyl-naphthylamine
PI	Propidium iodide
EtBr	Ethidium bromide
TSB	Tryptic soy broth
TSA	Tryptic soy agar
MH	Mueller–Hinton
MPCpr	Provisional mutant prevention concentration
